# Neglected spaces: UK general practice surgery waiting rooms

**DOI:** 10.3399/bjgpopen17X100641

**Published:** 2017-02-15

**Authors:** Gary Clapton, Lesley Reid

**Affiliations:** 1 Senior Lecturer, Social Work, University of Edinburgh, Edinburgh, UK; 2 Senior Health Promotion Specialist, Health Promotion Resource Centre, NHS Lothian, Astley Ainslie Hospital, Edinburgh, UK

## Introduction

Every day GP surgery waiting rooms are used by thousands of people, yet the experience of waiting and the nature of the waiting rooms in these surgeries has been neglected as a subject for critical attention. This article reports on a unique photographic research project conducted with GP waiting rooms as the research subject.

## The literature

Using the key phrases ‘healthcare’ and ‘waiting rooms’, and discounting those studies that addressed issues such as length of waiting times, the following databases were searched: CINAHL (Cumulative Index to Nursing and Allied Health Literature), Embase, Health Management Library, HMIC (Health Management Information Consortium), and PubMed. An iterative process was used to select 24 studies that were concerned with the way patients were affected by their surroundings while they waited to see a GP. Although not comprehensive, this literature search has found a broadly representative sample of the existing writings, the points of which are summarised below.

Few people look forward to waiting^1 ^and therefore the acknowledgement of patient anxiety and the value of stress-reducing ‘positive distractions’ is frequent. Positive distractions include views of nature, music, and visual art.^2^ It is argued that perceptions of the quality of care taken over the appearance of a waiting room can be a proxy for care taken over a patient’s health^3,4 ^and the literature has addressed seating arrangements,^1 ^smell,^5 ^decoration,^6 ^with a view to conveying a sense of ‘homeyness’ that that according to Goelitz and Kahn^7 ^‘helps the space feel more like a living room’. Dilani^8 ^sums up this approach by suggesting the adoption of a ‘salutogenic’ approach to health waiting spaces which focuses on ‘the factors that keep us well, rather than those that make us unwell’.

The waiting period is, in the words of Sherwin *et al,* ^9^a ‘key, yet neglected segment in health care’. A few writers have given thought to what Tsai and colleagues^10 ^in their study of a hospital out-patient waiting area, term the ‘atmospherics’ of place. Gillespie’s study of the relationship of architecture and a waiting room in a family planning clinic is one example^11 ^and another is that of Evans *et al*,^12 ^that explores the use of artwork in hospital waiting rooms in a discussion of the ‘therapeutic aspects of medical spaces’. Just as the waiting period is underexplored, the surroundings in which this happens are equally neglected. Neuwelt and colleagues^13^ have drawn attention to this and call for greater exploration of the ‘complex but under-recognised internal geographies’ of waiting rooms.

Finally, there was a lack of a discussion of the content (images and text) of the health education materials and general wall displays. One study discussed the presence of potentially stress-inducing displays such as alarming images of cancers.^14^

## Methodology

### Data collection process

Using the notion of ‘internal geographies’, this study's working research question was, ‘what factors within and around the GP waiting room, can determine or influence the patients’ waiting experience?’ Petersen and Østergaard^15 ^talk of how photos can be used to ‘spur new ways of looking upon things we otherwise do without paying particular attention’. To the authors' knowledge there has only been one other study that has used photographic means to ‘get at’ what it might feel like to wait in a health facility waiting area. This was undertaken in the US and the images were subsequently used to stimulate conversations with those who use the facilities.^2 ^Therefore, ‘new ways of looking upon’ waiting rooms seemed to offer the possibility of new insights.

The entire waiting areas were photographed, including the reading materials and layout, taking into account the items in the room and other aspects, such as official notices, reception desks, and TVs. Essentially, the researchers tried to ‘notice’ all that they could. In this process, the researcher and photographer became co-researchers, intuitively drawing one thing or another to the other’s attention as the work developed;^16^ for example, the existence (or not) of water coolers became an item of enquiry half way through. Likewise, the reception desks, access to them and their layout became a subject after noticing one difficult-to-engage-with reception. The latter dimension of the research process coincides with what Pauwels^17^ described as ‘researcher-generated imagery’. These data were collected when the waiting rooms were empty, often during lunch times or after standard appointment times had finished. In all, 23 waiting rooms and their surroundings (foyers and external entrances) were photographed in one Scottish city and an average of 40–60 minutes was spent in doing so.

Buchanan^18 ^notes that, ‘images may reveal more about the photographer than the photographed’ and Harper^19 ^remarks that ‘all images are socially and technically constructed’. Thus the research process, as for much social science research, though reflexive, cannot claim to be objective, nor repeatable. However, it is hoped that the transparent approach to methodology will allow the applicability of these findings to be assessed, and at the least, for the findings to provide a stimulant to discussion and further research.

### The ethnological dimension

The process of data collection meant that sounds (TV and radio), lighting (natural and artificial) and impressions (overall welcoming ‘feel’ or otherwise) were part of, and influenced, the process of the research. This analysis has omitted all but the factual, for example sensory information such as sound and lighting, however, inevitably, an element of bias formed by whether the researchers felt welcome or comfortable in a given place, or not, was present.

### Analytical methods

The approach to analysis was informed by Pauwel’s idea of photographs as ‘windows’ to the depicted world.^17 ^It is also acknowledged, as indicated above that this data set is a unique construction of our making and began with an exploratory search for patterns. Honigmann^20 ^writes: ‘Pattern recognition begins with the anthropologist's inspecting a series of things, field notes, or other fieldwork documents, including photographs and audiotapes, and abstracting from them one or more general features recognized in the event’. This was operationalised in two stages. The co-researchers involved in data collection were joined by the third member of the research team and each was given the task of viewing the images alone and making notes as to impressions and any emergent themes (patterns) in the data. The addition of the third member contributed a ‘cold read’ to the data given that she was not present during the photography. The three researchers then came together and jointly looked over the photographs and compared their first, individual, thoughts. Then using a collaborative, grounded theory approach^21^ the photographs were coded into a series of categories.

## Findings

Six key themes emerged and are described below.

### The unloved and the pleasant

The ‘unloved’ and ‘pleasant’ are subjective descriptions and these categories can be considered in the words of Robson^22 ^as having ‘latent content’; that is, content that is not manifest but implicit, the presence of which is the result of interpretation by the research team.

**Figure 1.**
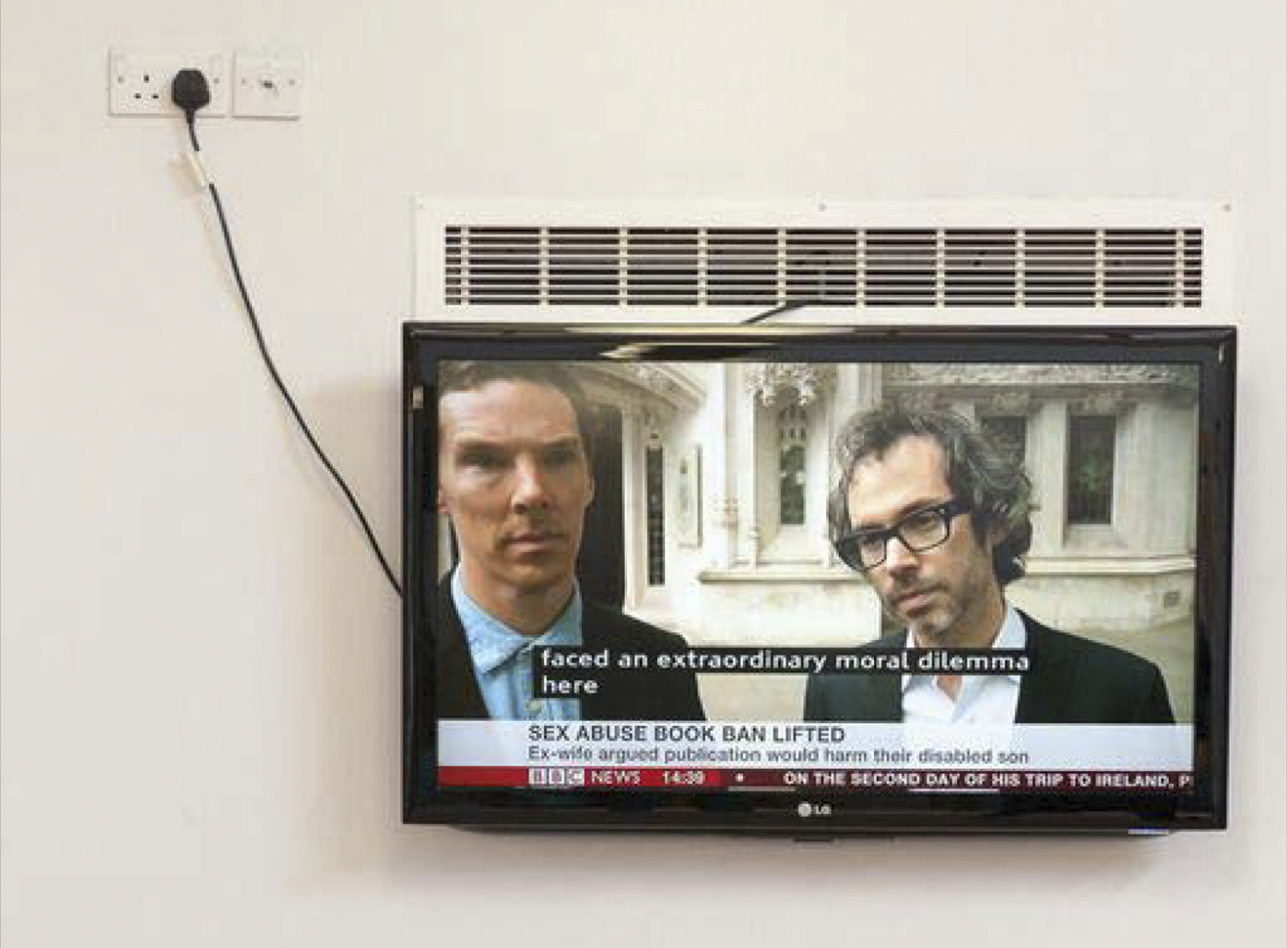
Rolling news.

- Pleasant: a minority of waiting rooms were striking in their inclusion of windows to gardens or greenery, lightness, and airiness. Waiting rooms in this category also included plants, fish tanks, individual touches such as book-lending libraries, local art, and other connections with the surrounding community, such as recipe books for sale. The word ‘homey’ was used to describe these more comfortable, welcoming waiting rooms.
- ‘Unloved’: waiting rooms in this category contained TVs that were either switched off, switched to static, unplugged or featured sound-off ‘rolling’ news; radios playing ‘muzak’ (such as ‘Drive-time’ radio or pop) and had other negative features such as ‘Speak Here’ grilles to communicate with the receptionist (Figure 1).

**Figure 2.**
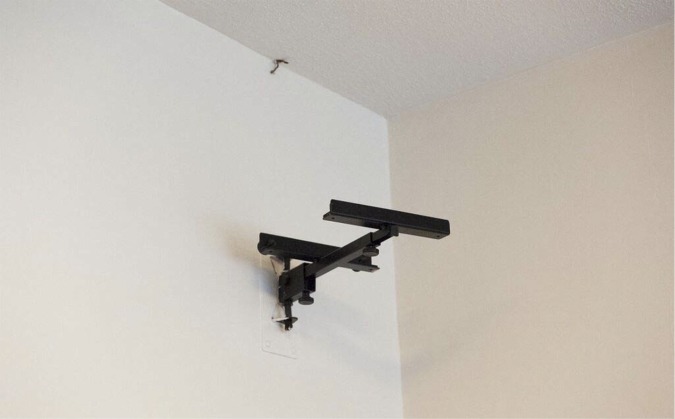
Empty TV cradle.

Other unpleasant aspects were empty TV cradles and obvious CCTV cameras. Less visually striking but contributory to a general unloved air was the use of old sellotape or blue-tack to pin up leaflets (Figure 2).

**Figure 3.**
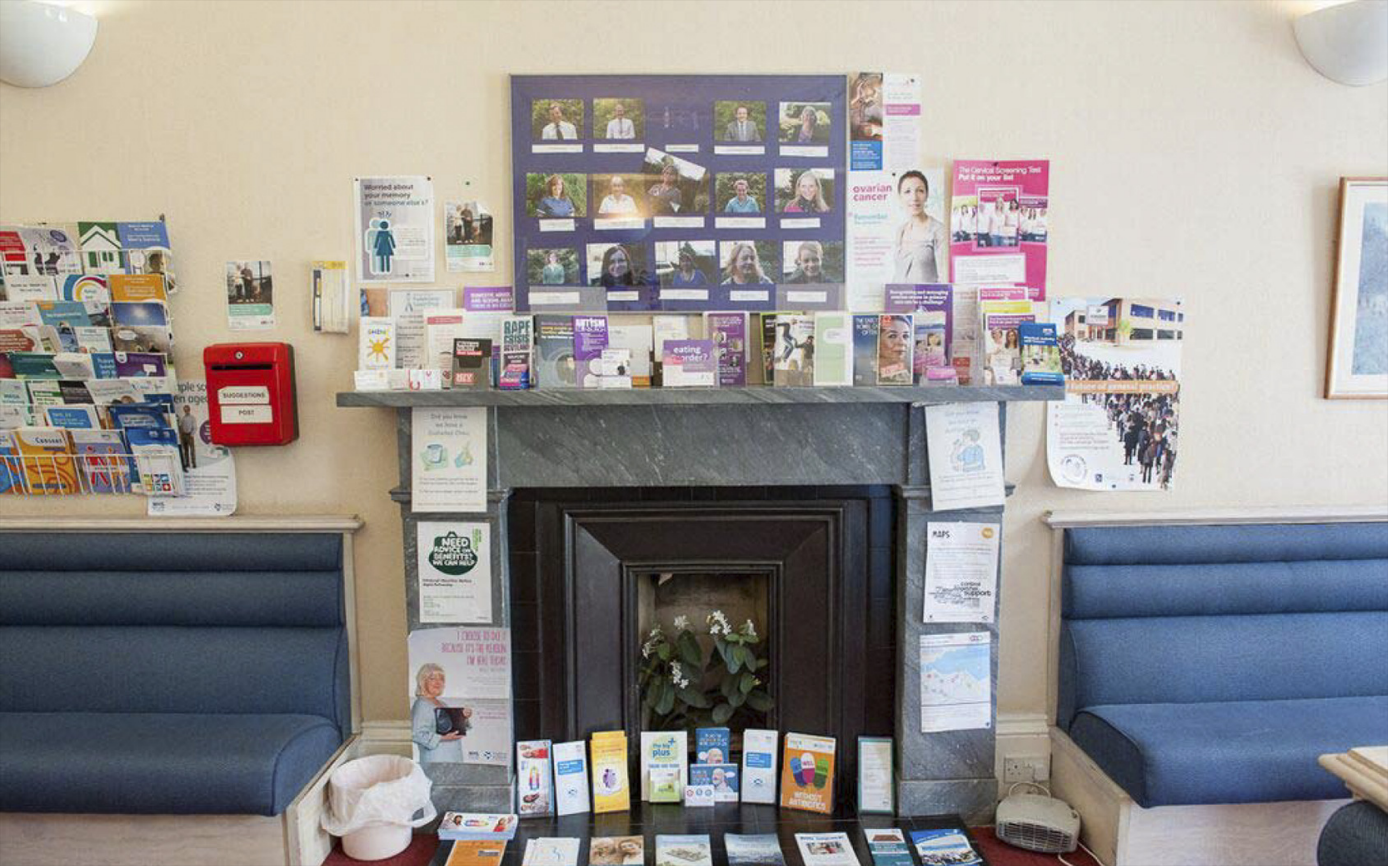
Leaflet clutter.

There were a number of approaches to the distribution and dissemination of health-promotion materials. Predominant was what was termed ‘leaflet clutter’ with every available surface in one waiting room being covered with stacks of health or sickness-related information (Figure 3).

Some posters were placed in unreadable areas, (such as high on walls). One praiseworthy approach confined leaflets and other promotional materials to one designated theme each month in rotation; for example, mental health month.

Some receptions were ‘homey’ and contained less comforting aspects such as leaflet clutter, hard-to-see receptionists and intrusive noise. Some ‘new-builds’, with airiness, big spaces, and rows of seats were charmless ('bus stations and shopping malls'), yet they featured coordinated colour schemes, ergonomically designed fronts and interiors, pleasant art on the walls, fish tanks, and facilities such as water coolers.

### Nurturing and constructive use

This category emerged via the identification of images that depicted services for patients (or not). A minority of locations offered water coolers; one offered two fizzy drinks dispensers, one of which was out of order; and one new surgery was adjoined by a coffee shop. Such non-medical services in a medical setting seem to be a way of bridging gaps between the world of the waiting room and the rest of a service user’s existence. Helping oneself to water or buying refreshments (even though they are fizzy drinks) may help mitigate any sense of lack of agency.

One example of a ‘while-you-wait’ questionnaire was found and three surgeries used digital facilities such as log-in screens. One surgery had made available a walk-in pod with a self-weighing and blood pressure check machine which sent a patient's results straight into their medical file.

### Reading distractions

As distinct from health or sickness-related reading, there was a range of approaches to the provision of fiction and non-medical materials. Although there were very few daily newspapers in sight, some surgeries had provided small libraries. One had *Poems in the Waiting Room*, a collection of works curated to offer distraction.

Although there was some traditional male-related reading (*Car Golf Monthly* magazines), the bulk of reading materials in most waiting rooms were ‘women’s’ magazines such as *Bella* and *Take A Break*.

### Layout

**Figure 4.**
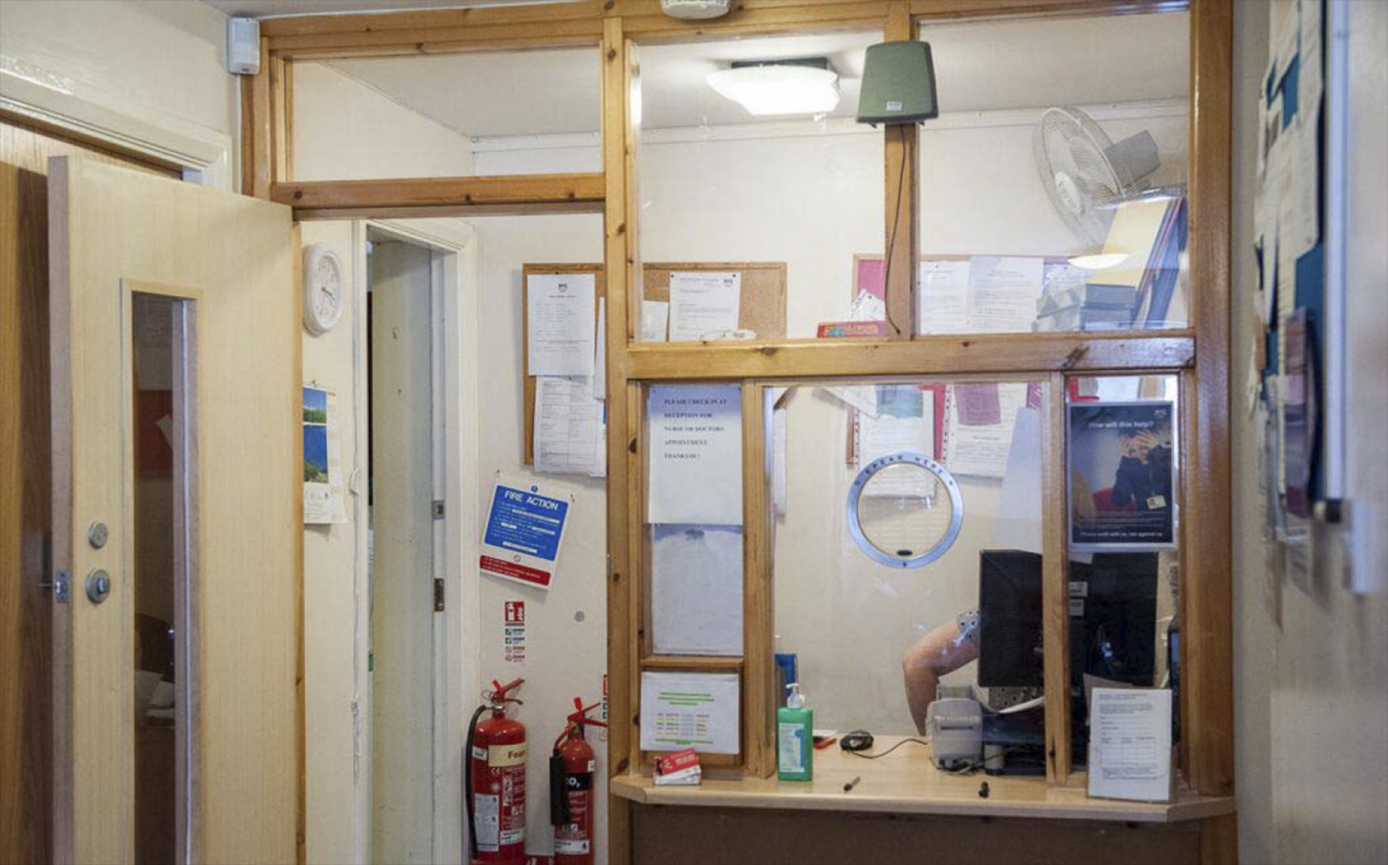
Obscured receptions.

In some receptions, visual access to the receptionist was obscured. This was often where PC screens reached well above the level of the counter. ‘Over-decoration’ of the reception was also found (Figure 4).

**Figure 5.**
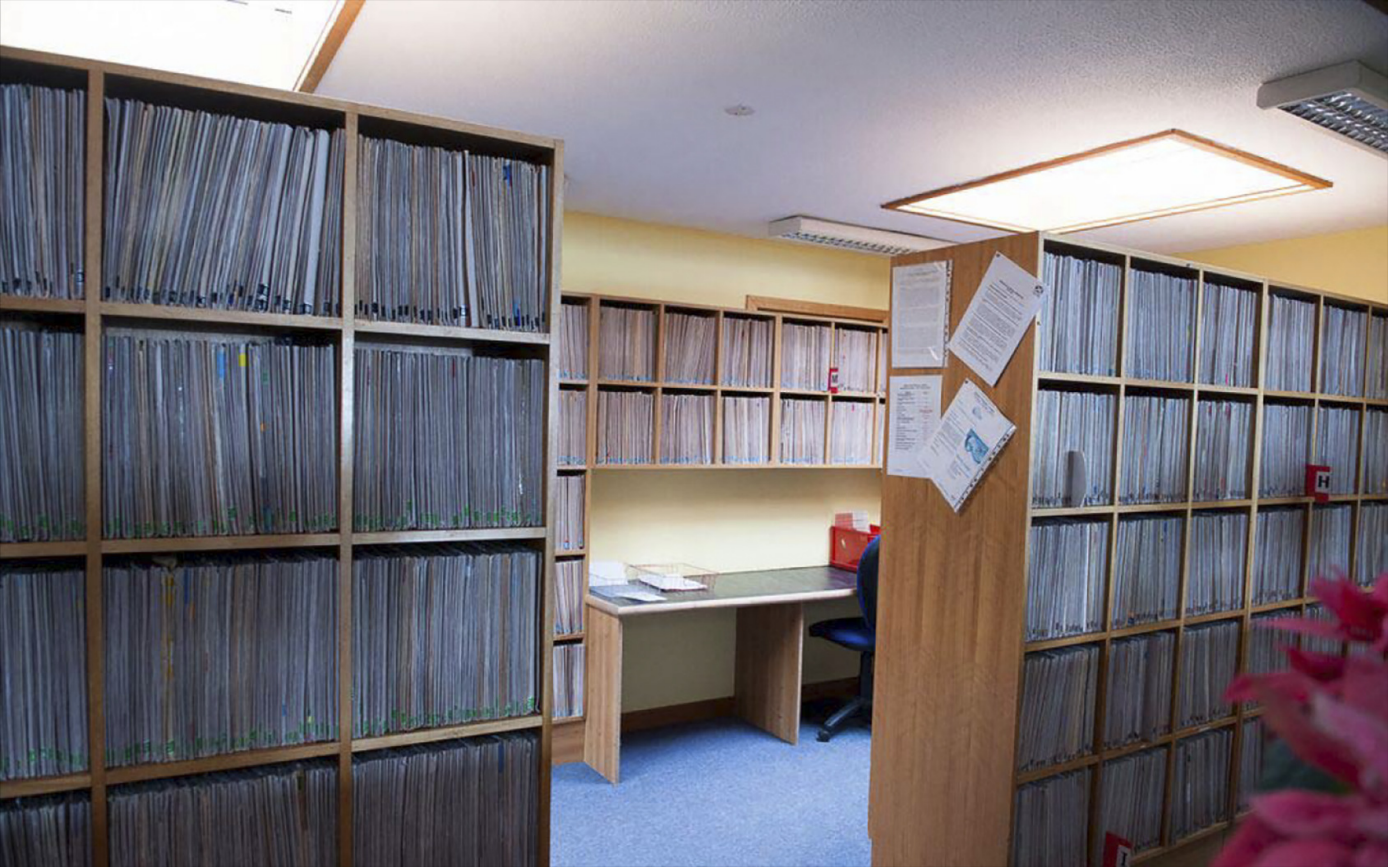
Visible banks of case files.

The ‘Speak-Here’ grille in one surgery and this together with obscured receptions, gave off a defensive tone. In other instances, the layout of the waiting room and sight lines meant that banks of files were on display and this lent an impersonal quality to the visual environment (Figure 5).^23^

### Addressing the public

**Figure 6.**
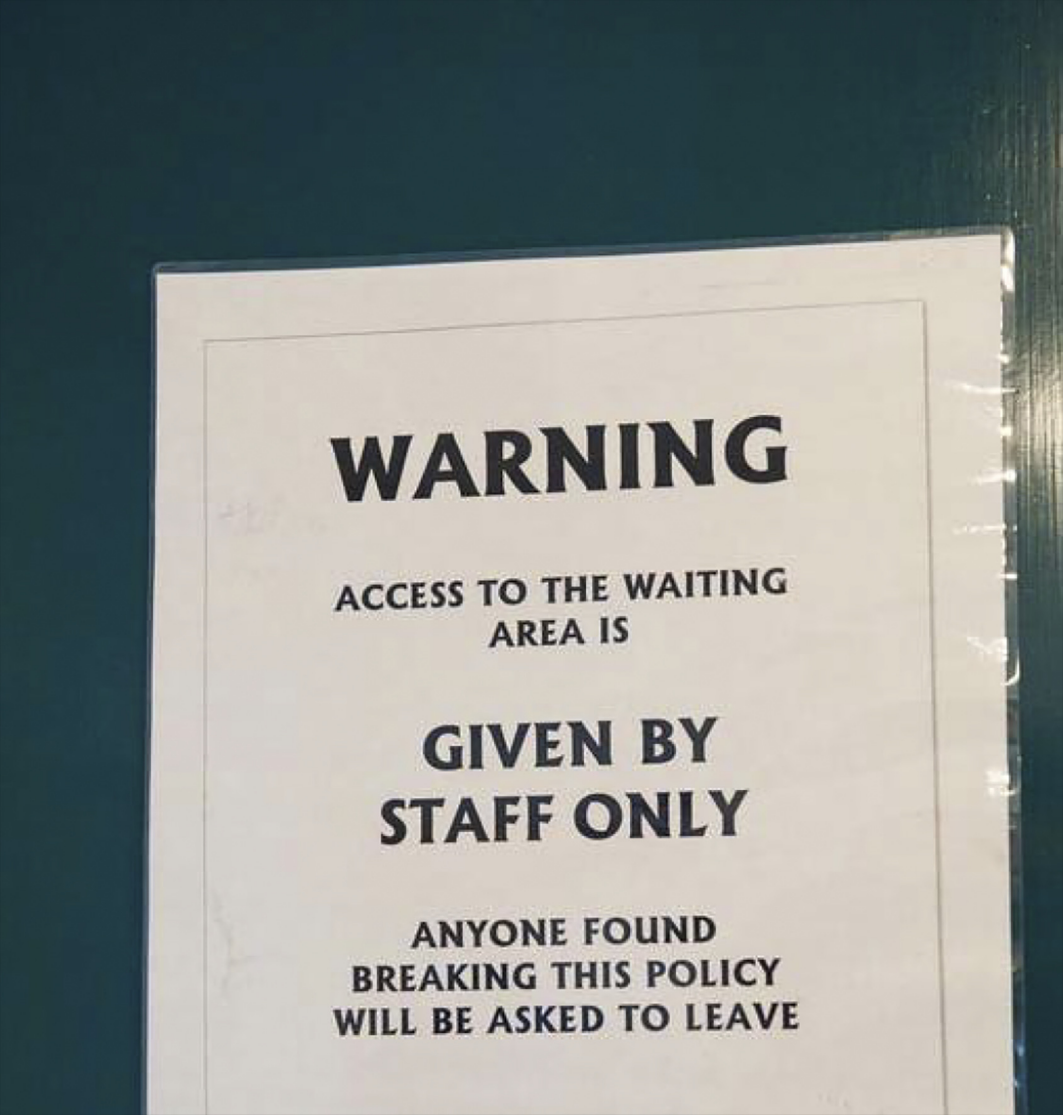
Public notices.

This category developed as a result of the many instances of public notices. Many of the various messages were instructional in tone, talking at the reader with a list of dos and don’ts as in 'could all patients please refrain from …' in cod-polite speech (Figure 6).

Others reminded the reader that 'CCTV surveillance was in operation'. In one location, information of the ‘dos’ and ‘don’ts’ type was placed high up on the walls and was in small print. It was also considered whether ‘No smoking’ signs were still necessary.

### Worry

**Figure 7.**
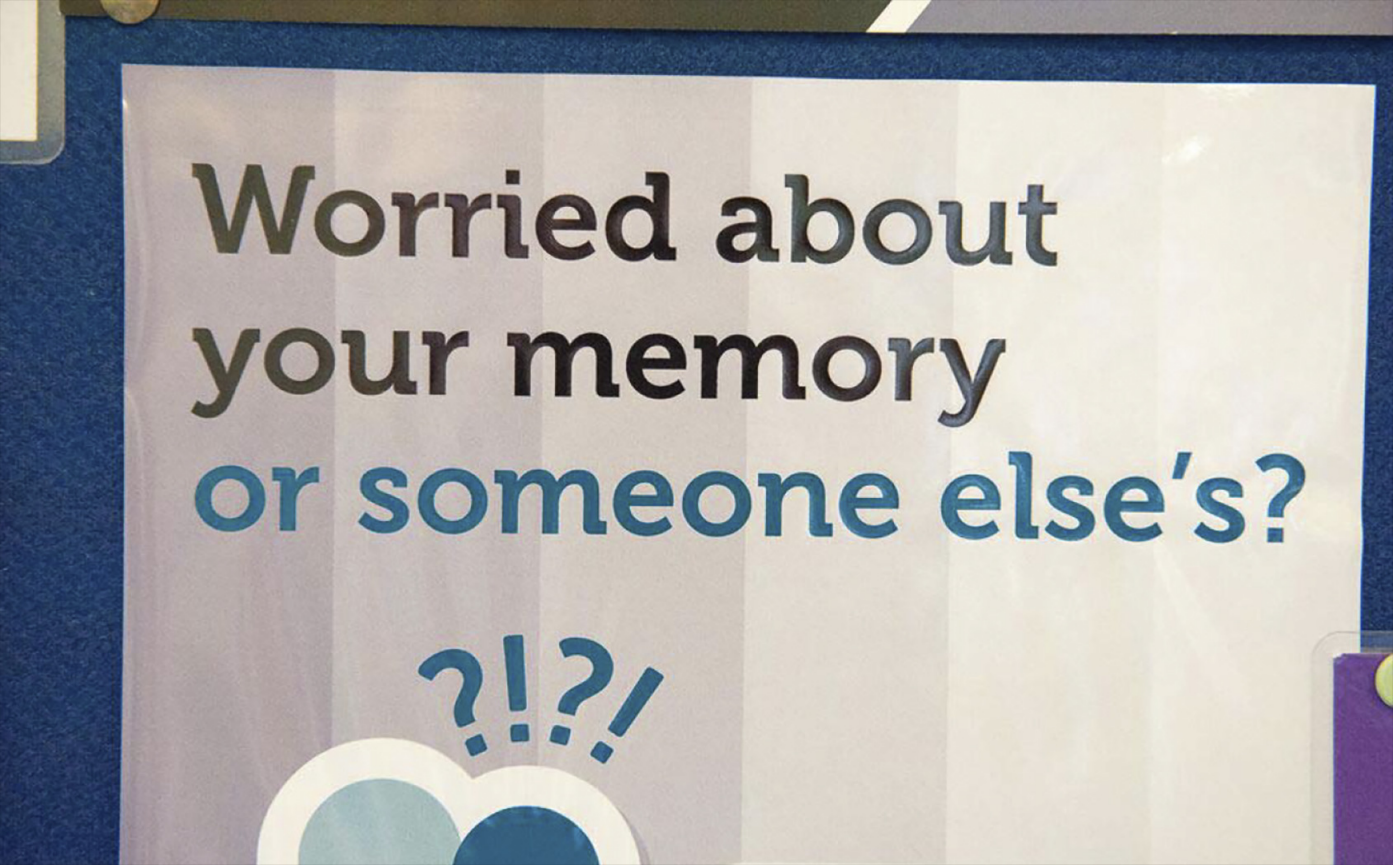
Worry.

A feature of the data gathering was that as the photography proceeded, the two researchers involved began to notice the many times that posters and leaflets in the waiting rooms featured the words ‘worry’, ‘anxious’, ‘concern’ or ‘problem’ (Figure 7).

**Figure 8.**
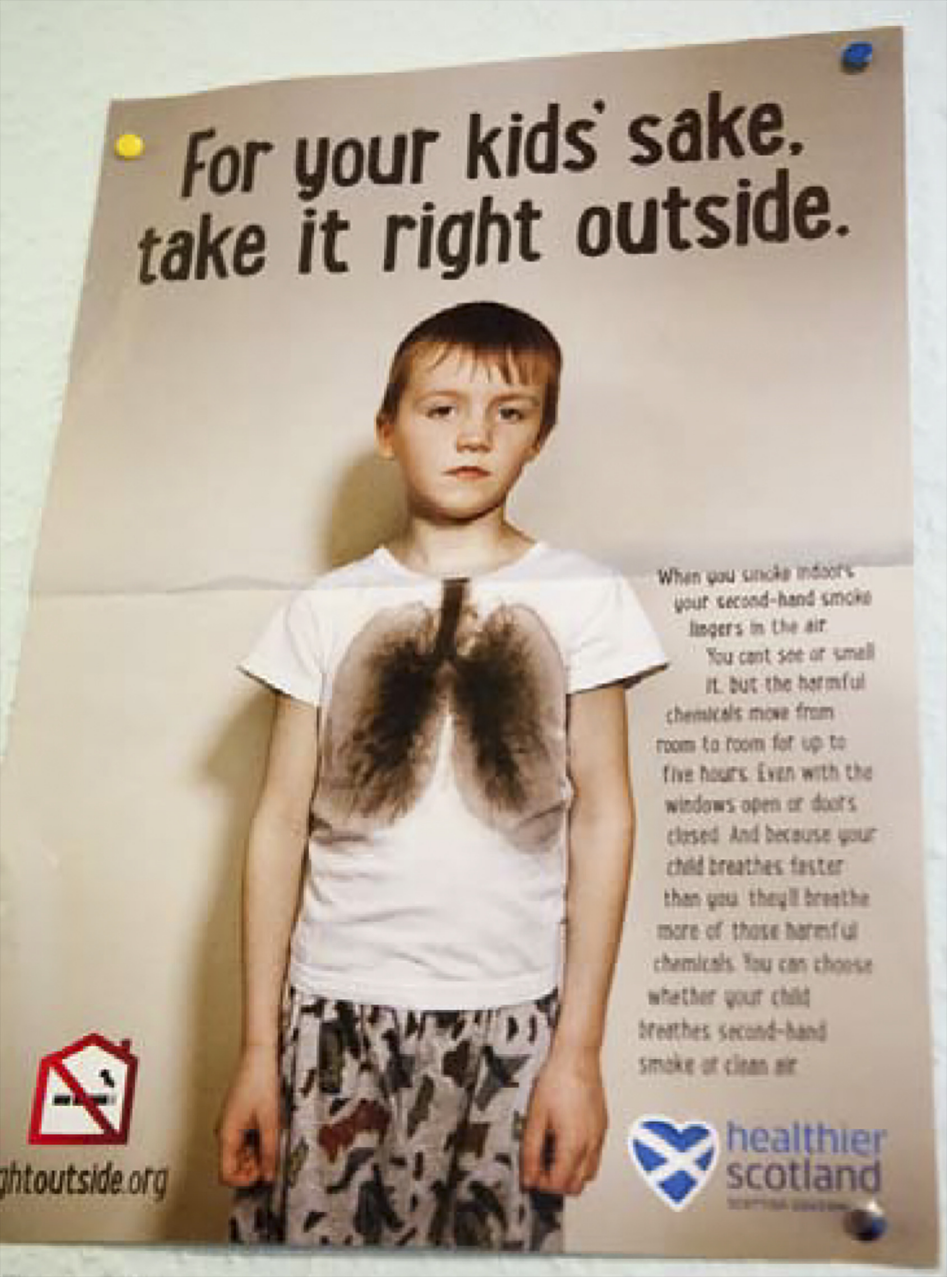
Shock.

This chimes with a concern expressed in the study by Gignon *et al* about the possibility that some display materials could provoke anxiety due to their ‘shock content’ (Figure 8).^24^

## Discussion

### Summary

This research found attention to appearance in waiting rooms to be widespread, some of it less successful than others with ‘homey’ waiting rooms being adversely affected by stacks of leaflets on every surface. Elsewhere, new facilities with plenty of light and air were somewhat compromised by out-of-order drinks machines, the sight of medical files, and reception areas with lines of sight obscured by computer monitors. There was interactive use of the waiting time, for example the possibility of having service-users participate in their own assessments via while-you-wait questionnaires and weighing scales, in only two of the 23 surgeries visited. A neglect of consideration of how surroundings influence the overall waiting experience is the main finding.

### Strengths and limitations

This study had a subjective dimension. While ‘objective’ and verifiable findings are the goal in health-related research, these findings are inferential in nature and not intended to prove or disprove, they are offered in the spirit of making the ‘familiar strange’ so as stimulate discussion on an everyday but overlooked space and place.

Because this subject was the waiting room space, the experiences and views of patients or staff were not examined — the Picker Institute report provides a useful account of UK staff and patient views^3^ — and it is worth recording that empty waiting rooms are different from those that are filled with people. Nor has this study specifically addressed the waiting room in relation to the differing experiences and needs of particular age groups, such as older people, teenagers, toddlers, and babies. And finally, this sample was not large enough to make generalisations.

### Implications for practice

This study has found a neglect of attention to the surroundings that may influence the waiting experience in GP surgeries. If this time is framed as part of the patient journey before the encounter with their GP, then what affects patient mood and receptiveness during this period becomes significant. What follows are some concrete suggestions that arise from this research, taking the cue from Sherwin *et al* ^9 ^to think about the waiting period as an opportunity.

#### The ‘unloved’: deal with the clutter of health information

The materials sent to surgeries and displayed can often be overwhelming and confusing. The selection and display of materials can also be selective in nature. Health messages which are backed up by expensive mass media advertising can be lost among other materials. Incoming knowledge can be curated to avoid this; that is someone with responsibility for quantity, content, and timing of displays with the idea that less is more (for example, one rotating theme per month is better than the present free-for-all); this can be achieved using hard copy or a display screen.

#### Maximise nurturing and reduce feelings of loss of control, passivity and anxiety: greet, assure, and boost agency

There are a few key messages that need to be known about the surgery and how it works in order to help someone know they’ve come to right place, and are welcome. A visible screen would be a useful way to do this. Such a screen, alongside the standard biographical details of staff, could also list other key messages about facilities and contact points.

Some interesting suggestions have arisen in the course of this study. These include greater involvement in the process of seeking and receiving medical advice, for instance the provision of paper pads with questions on them along with pencil and pens to help people think through how they are going to introduce the subject, and to list the questions they want to ask medical staff. And what about the ability to access one’s case notes? This is technically challenging, yet a password-protected system may take some of the mystique out of the visit, provide both a record and reminder of matters to be covered or raised and those already satisfactorily addressed, and reduce the potential for loss of agency. Such an approach would be in keeping with recent writing on co-production in health services delivery that values the expertise that the patient brings to the meeting between themselves and the GP.^25^

#### Reading distractions: use the platforms

It is important to acknowledge that non-health related reading has probably been superceded by patients’ use of social media while they wait. Surgeries should consider providing access to scannable relevant non-health and health-related literature, including *Poems in the Waiting Room* which are more likely to be read online.

#### Layout and waiting room environment

There are a number of points about the environment that occur in the literature that have also been found in this study. These include the reduction in ‘instructions’ to the public, the avoidance of negative distractions such as muzak and rolling TV news and the promotion of positive ones such as fishtanks, aspects of, and access to, nature, and diverse non-health-related reading materials. The waiting room environment should also offer services such as water and consideration should be given to providing free WiFi, wall-fixed iPads and social media charging stations. The reception area, such as counters, should be reviewed to ensure clean sight lines that do not include files and are relatively clutter-free.

#### Worry: the need for balance

A balance needs to be struck between messages about health that are hard-hitting and contain a shock element — for example, images of diseased lungs, a favoured advertising tactic for a number of years — and other ‘softer’ forms of persuasion.

## Conclusion

In conclusion, it is worth repeating that empty rooms were photographed, although many of these findings were echoed in the existing literature. Going forward, any discussion of changes must have the participation of staff.^26 ^ Partnerships or focus groups involving health centre staff and users have proved successful in improving aspects of the waiting experience, such as the provision of refreshments.^27 ^Such ‘horizontal’ collaborations can also boost patient agency. The belief that control can be exerted over environment has an organic connection to a belief in control of one’s good health.
